# Reoperative Aortic Root Replacement in Patients with Prior Aortic Valve, Root Replacement, or Arch Replacement Surgery: A Single-Center Experience

**DOI:** 10.1055/a-2542-4443

**Published:** 2025-04-08

**Authors:** Toyokazu Endo, Jaimin R. Trivedi, Priyadarshini Chandrashekhar, Michele Gallo, Erin M. Schumer, Biran Ganzel, Mark S. Slaughter, Siddharth Pahwa

**Affiliations:** 1 Department of Cardiovascular and Thoracic Surgery, University of Louisville School of Medicine, Louisville, Kentucky; 2 University of Louisville School of Medicine, Louisville, Kentucky

**Keywords:** aorta, aortic valve, aortic root, aortic conduit, reoperative, perioperative outcomes

## Abstract

This case series evaluated the characteristics and outcomes of reoperative root replacement in patients with prior aortic valve replacement (AVR), aortic root replacement (ARR), or ascending or hemiarch replacement (AoR) from our single institution from 2014 to 2023. A total of 55 (prior surgery: 38 AVR, 5 ARR, and 12 AoR) patients were identified and indication for reoperation included valvular stenosis, endocarditis, aneurysm, and dissection. Perioperative mortality was 10.9% (6 patients) (inpatient complications: 2 stroke, 4 bleeding event, 2 renal failure, and 21 prolonged vent).

## Introduction

Aortic root replacement (ARR) is generally safe, with a mortality rate below 5%. However, as the current patient population ages, the need for reoperative root replacement increases. Reoperative procedures are technically challenging and pose higher risk due to issues like sternal reentry, coronary ostial mobilization, and coronary artery reimplantation, among others. 1 A limited number of studies have evaluated clinical outcomes following reoperation, especially after a prior aortic valve or root replacement surgery. Most current literature focuses on redo sternotomies or reoperative root replacement in patients with any prior cardiac surgery history. 2 3 This study evaluates the characteristics and outcomes of reoperative ARR in patients with prior aortic valve replacement (AVR), ARR, or aortic conduit surgery (i.e., ascending aorta or hemiarch replacement).

## Methods

A single-center cardiac surgery database from January 2014 to June 2023 was used to identify patients undergoing reoperative ARR. All patients had prior aortic valve, aortic root, ascending aortic, hemiarch, or any combination of these surgeries (IRB approval: 16.0210). Patients undergoing other prior cardiac procedures such as other valve repair/replacement, coronary artery bypass grafting, descending thoracic aortic surgery, or aortic arch surgery were included. Patient characteristics and outcomes were evaluated using descriptive statistics (median [interquartile range]), or *N* (%). Two groups were created, one with prior AVR and one with prior ARR or aortic replacement or hemiarch replacement (AoR). Patient characteristics and outcomes were compared using the chi-squared test and the Kruskal–Wallis test with an α of 0.05. Patients' follow-ups were obtained based on their most recent patients' encounters recorded in the electronic health record.

## Results

### Patient Characteristics

A total of 55 patients were identified during our study period ( Table 1 ). A total of 38 patients had a prior AVR (69%) and 17 had a prior root replacement, ascending, or hemiarch replacement (31%). The reasons for reoperation included endocarditis (14 [25.5%]), aneurysm formation (18 [32.7%]), and valve stenosis (15 [27.3%]). The median years from prior aortic surgery was 8, with an interquartile range (IQR) of 2 to 14 years. The median age of our patients was 63 (56–70). More than half (61.8%) were male. Median body mass index and body surface area were 28 (25–32) and 2 (1.7–2.1), respectively. Nine patients (16%) had a history of moderate chronic lung disease, 31 (56%) had a history of cigarette use (or current), and 13 (24%) had a Type 2 diabetes. Most of our patients had hypertension (82%), and 20% had suffered prior myocardial infarction.

**Table 1:** Patient Characteristics of Redo Aortic Root Replacement

Variables	Total	Prior AVR	Prior AR	*p* -Value a
*N*	55	38	17	
Prior aortic surgery				
Prior AVR	38 (69.1)	38 (100%)	0 (0)	
Prior root replacement	5 (9.1)	0 (0)	5 (29.4)	
Prior ascending aorta/or hemiarch replacement	21.8)	0 (0)	12 (70.6)	
Concomitant procedure				0.6223
CABG	3 (5.5)	3 (7.9)	0 (0)	
Other valves	5 (9.1)	4 (10.5)	1 (5.9)	
Age	63 (56–70)	63 (56–70)	62 (56–69)	0.8769
Gender (male)	34 (61.8)	22 (57.9)	12 (70.60)	0.3706
Race (White)	51 (94.4)	35 (94.6)	16 (94.1)	0.9433
BMI	27.9 (24.5–31.5)	27.9 (24.5–31.3)	27.2 (24.9–35.2)	0.9419
BSA	1.97 (1.74–2.14)	1.91 (1.71–2.09)	2.05 (1.85–2.15)	0.2328
Risk factors				
Chronic lung disease (≥moderate)	9 (16.4)	6 (15.8)	3 (17.7)	0.8634
Smoking Hx (current or former)	31 (56.4)	21 (55.3)	10 (58.8)	0.8057
Hx HTN	45 (81.8)	30 (79)	15 (88.2)	0.4092
Prior MI	11 (20)	8 (21.1)	3 (17.7)	0.7704
T2DM	13 (23.6)	11 (29)	2 (11.8)	0.1657
Renal failure (dialysis)	3 (5.5)	3 (7.9)	0 (0)	0.2335
Hematocrit	37.2 (29.6–40.3)	37.3 (29.1–40.3)	33.4 (29.8–40.3)	0.8919
Creatinine	1.01 (0.86–1.43)	1.00 (0.82–1.43)	1.01 (0.95–1.33)	0.4496
EF	55 (45–60)	53 (45–60)	60 (55–60)	0.142
Years from prior surgery	8 (2–15)	8 (2–15)	8 (4–15)	0.7977
Indication (reoperative)				0.3718
Valvular stenosis	15 (27.3)	13 (34.2)	2 (11.8)	
Endocarditis	14 (25.5)	8 (21.1)	6 (35.3)	
Aneurysm	18 (32.7)	12 (31.6)	6 (35.3)	
Dissection	5 (9.1)	3 (7.9)	2 (11.8)	
Other	3 (5.5)	2 (5.3)	1 (5.9)	
Operation urgency				0.3135
Elective	27 (49.1)	21 (55.3)	6 (35.3)	
Urgent	24 (43.6)	14 (36.8)	10 (58.8)	
Emergent	4 (7.3)	3 (7.9)	1 (5.9)	
Cross-clamp time	130 (104–144)	128 (93–151)	132 (120–141)	0.7746
Bypass time	171 (145–206)	171 (139–208)	167 (152–196)	0.8275
Outcome variables				
In-hospital mortality	6 (10.9)	3 (7.9)	3 (17.7)	0.2837
POLOS	8 (6–11)	9 (6–15)	7 (6–10)	0.1101
Stroke	2 (3.6)	2 (5.3)	0 (0)	0.3353
Bleeding	4 (7.3)	3 (7.9)	1 (5.9)	0.7906
Renal failure	2 (3.6)	2 (5.3)	0 (0)	0.3353
Prolonged vent	21 (38.2)	19 (50)	2 (11.8)	0.007

Abbreviations: AR, aortic root, ascending or hemiarch replacement; AVR, aortic valve replacement; BMI, body mass index; BSA, body surface area; CABG, coronary artery bypass grafting; EF, ejection fraction; HTN, hypertension; IQR, interquartile range; MI, myocardial infarction; POLOS, postoperative length of stay; T2DM, Type 2 diabetes.

a *p* -Value: Statistical comparison between AVR and AR.

### Operative Outcomes

The median cross-clamp time was 130 minutes (104–144), and the total time on cardiopulmonary bypass was 171 minutes (145–206; Table 1 ). The operative mortality was 10.9% (6). Median postoperative length of stay ranged from 6 to 11 days (IQR), with a median length of 8 days. Two patients suffered a stroke while inpatients (4%). Four patients required a return to the operating room for postoperative bleeding (7%), and two patients (4%) developed renal failure that required dialysis. A total of 38% of patients had a prolonged duration on the ventilator beyond 24 hours.

Of the six operative mortality, one suffered from uncontrolled postoperative bleeding, three were in irreversible cardiogenic shock that required multiple inotropic agents as well as venoarterial extracorporeal membrane oxygenation, one suffered from postoperative seizures of unknown etiology (unable to wean off ventilator, not following commands, and evidence of diffuse axonal injury without evidence of embolic or hemorrhagic cerebral vascular accident, and one went into acute respiratory failure that required prolonged ventilation. All of the patients were withdrawn from life support and were initiated comfort measures.

### Follow-up

Of the 55 patients, 42 patients had follow-up data available. The remainder of the patients' data were unavailable due to loss to follow-up, patients were moved out of state, or data not uploaded onto the electronic health record (EHR adopted in 2018 at our institution and some data may have not been uploaded from paper chart to the EHR). The median follow-up time was 3.4 years (range: 21 days to 10 years). At the 1-year mark, two patients required a late pacemaker placement. One patient required extensive endovascular stent placement for thoracic, abdominal, and iliac artery dissection. One patient required a kidney transplant. All follow-up computed tomography scans did not demonstrate any complication from the root replacement, and no patient required another cardiac operation.

There was a total of four deaths among the 42 patients in which follow-up data were available. The other two died of malignancy-related complications.

## Discussion

There is limited knowledge regarding outcomes following reoperative ARR after prior aortic surgeries. From our single-center database (2014–2023), we identified 55 patients who underwent reoperative ARR, showing an operative mortality of 10.9%. It is challenging to state the true risk of root replacement in patients with prior aortic surgeries in the current literature, given the majority of the studies include sternotomies as well as other cardiac operations as redo. In these reports where various other procedures other than the aortic region were included, the operative mortality was 12.1 to 14.3%. 2 3 However, these evidence suggest that redo-root replacement carries similar mortality risks compared with first-time root replacement.

The studies on true root replacement are scarce. However, there is one series in the literature that reports a true root replacement operative mortality rate of 14.7% (27/184). 4 True root replacement is infrequent; thus, in our study, we included other aortic procedures as inclusion. Like the reports similar to ours, we share similar in-hospital mortality compared with other studies that include previous aortic valve, root, or ascending arch replacement. Szeto et al 5 in a series of 156 patients (the majority being previous AVR) from 1995 to 2006 reported a hospital mortality of 11.5%. Similarly, Huebner et al, 6 in their series of 130 patients (50 with previous ARR), reported an operative mortality of 10%. Although our case series are limited in numbers, redo ARR has acceptable perioperative risks. With the growing age of our population, further reports from other institutions will help differentiate the risks associated with each procedure.
